# Contribution of Continuous Virtual Monitoring to Hospital Safety, Quality, and Value of Care for COVID-19 Patients

**DOI:** 10.1089/tmj.2022.0061

**Published:** 2023-02-06

**Authors:** Corey Morrow, David Wheeler, Mary Dooley, Emily Warr, Ryan Kruis, Kathryn King, Jillian Harvey, Kit N. Simpson

**Affiliations:** ^1^ Healthcare Leadership and Management, College of Health Professions, Medical University of South Carolina, Charleston, South Carolina, USA.; ^2^ Center for Telehealth, Medical University of South Carolina, Charleston, South Carolina, USA.; ^3^ Department of Pediatrics, College of Medicine, Medical University of South Carolina, Charleston, South Carolina, USA.

**Keywords:** telemedicine, telehealth, continual virtual monitoring, time-driven activity-based costing, COVID-19, cost savings

## Abstract

**Introduction:**

The rapid onset of the COVID-19 pandemic increased hospital admissions and shortages for personal protective equipment (PPE) used to slow the spread of infections. In addition, nurses treating COVID-19 patients have time-consuming guidelines to properly don and doff PPE to prevent the spread.

**Methods:**

To address these issues, the Medical University of South Carolina repurposed continuous virtual monitoring (CVM) systems to reduce the need for staff to enter patient rooms. The objective of this study was to identify the economic implications associated with using the CVM program for COVID-19 patients. We employed a time-driven activity-based costing approach to determine time and costs saved by implementing CVM.

**Results:**

Over the first 52 days of the pandemic, the use of the CVM system helped providers attend to patients needs virtually while averting 19,086 unnecessary in-person interactions. The estimated cost savings for the CVM program for COVID-19 patients in 2020 were $419,319, not including potential savings from avoided COVID-19 transmissions to health care workers. A total of 19,086 PPE changes were avoided, with savings of $186,661. After accounting for cost of the CVM system, the net savings provided an outstanding return on investment of 20.6 for the CVM program for COVID-19 patient care.

**Conclusion:**

The successful and cost saving repurposing of CVM systems could be expanded to other infectious disease applications, and be applied to high-risk groups, such as bone marrow and organ transplant patients.

## Introduction

The rapid onset of the COVID-19 pandemic increased hospital admissions and shortages for personal protective equipment (PPE) used to prevent the spread of infections. High PPE demand led to increased PPE prices, and scarcity, and some products increased by >1,000%.^1^ Process improvement to reduced use of PPE would help with the shortage and reduce hospital costs.

Nurses caring for COVID-19 patients have additional time-consuming responsibilities to prevent spreading the virus. When working with COVID-19 patients, nursing staff must don and doff PPE every time they enter a patient's room.^2^ Use of PPE is a routine infection control requirement when entering a contagious patient's room, but the pandemic greatly increased the need for this precaution. PPE is commonly used for patients with other contagious infections such as *Clostridioides difficile* (CDI) and Methicillin-resistant *Staphylococcus aureus*.

However, the hospital prevalence is comparatively much lower (i.e., 1.47 CDI cases per 1,000 patients) than the highly prevalent COVID-19.^3^ Our hypothesis was that using continuous virtual monitoring (CVM) to reduce the nursing time spent on PPE without compromising patient and staff safety would lead to cost savings.

CVM allows for remote monitoring and communication with patients. A common use of a CVM system is to replace an in-person sitter assigned to observe high-risk patients to prevent falls, self-harm, or other dangerous behaviors. This system allows for a higher patient-to-staff ratio that reduces costs without sacrificing patient safety.^4^ The Medical University of South Carolina (MUSC) uses a CVM system that was initially developed and customized to ensure reliable and high-quality continuous audiovisual streaming, robust security/privacy protections, and ease of use for clinicians. During 2019, the CVM system was used for monitoring patients at risk for falls and other behavioral safety incidents for 4,721 inpatient days on 23 inpatient units and in the emergency department.

As part of COVID-19 preparation in March 2020, MUSC rapidly deployed its CVM system, with modifications to ensure patient privacy, to inpatient negative pressure rooms for patients either awaiting test results or with confirmed COVID-19 infection. The presence of CVM in a room assured timely response to patient needs and questions, reduced risk of transmission of the virus to staff, and preserved scarce PPE. The objective of this study was to identify cost impact and PPE usage differences associated with the CVM program for COVID-19 patients.

## Methods

From a hospital cost perspective, the CVM program was focused on reducing nursing time and PPE costs per patient. We used a time-driven activity-based costing (TDABC) approach to determine time and costs saved by implementing CVM.^5^ This approach determines the cost of an activity by using process analysis to estimate the minutes spent of all tasks involved in that activity. In this study, patient monitoring/nurse call answering was the *activity*, and tasks such as donning and doffing equipment were the *required tasks*. We used a hospital cost perspective in this analysis and recorded resources used to which we applied standard cost values.^6^ The project was classified by the institutional review board as a quality improvements project that did not require patient-informed consent.

### COSTS OF TIME

A videorecording on guidelines for use of PPE by the Centers for Disease Control and Prevention (CDC) was viewed and time in minutes for the donning/doffing process was recorded using a stopwatch. There were in total 10 min timed for donning (4 min) and doffing (6 min). The times included hand washing in the donning/doffing process. For easier calculation, these 10 min were converted into 0.167 h of time (10/60). In-person nursing interactions averted were counted by the number of times the CVM–patient call system was used without an escalation to need an in-room encounter.

Next, salary costs were calculated for nursing staff. According to the U.S. Bureau of Labor and Statistics, the national average salary for nursing staff is $45 per hour.^7^ The costing approach used assumptions of productivity (80%) and fringe benefit (30% of salary) levels that are generally accepted for TDABC studies.^8^ After adding 30% to the base salary for fringe benefits, the loaded salary average is $58.50. The salary average was adjusted to reflect a typical productivity standard of 80% for an effective hourly production salary of $73.13. The PPE donning/doffing activity time (0.167 h) was multiplied by the number of in-person interactions averted and the staff effective hourly salary ($73.13) to determine the costs averted from time saved on PPE use.

### COSTS OF EQUIPMENT

Each CVM system contained an interactive telehealth monitor and portable cart. The cost of each CVM system was $4,500 amortized over 3 years ($1,500/year). This equals $4.11 per day per CVM system. An additional $10.00 per day was added to capture cost of daily cleaning of the CVM system for a total of $14.11 per day. Owing to patient isolation, only one patient has access to a CVM system at one time.

**Fig. 1.**
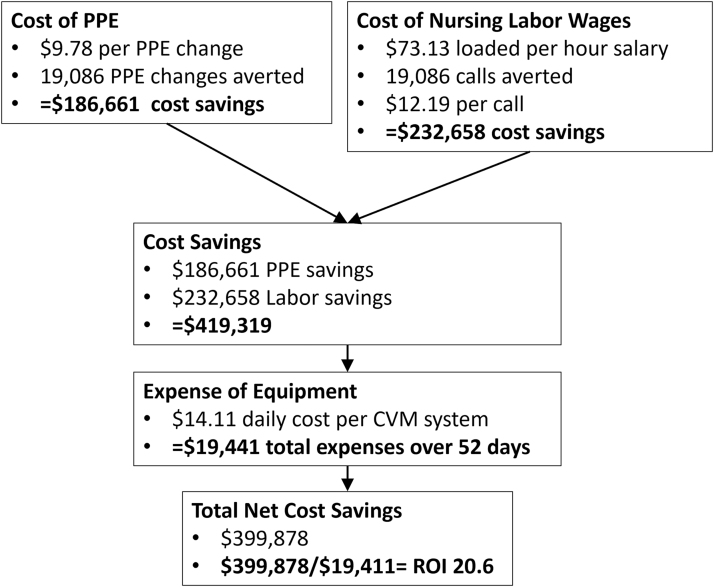
Cost savings analysis. CVM, continuous virtual monitoring; PPE, personal protective equipment; ROI, return on investment.

Next, we constructed a spreadsheet to estimate the cost for the reduction in PPE use. Staff could use two types of gloves (vinyl and nitrile) and masks (3-ply and N95). Mean costs were calculated for gloves ($0.08 per pair), masks ($4.25 per mask), gowns ($5.00), and face shields ($0.45) by adjusting costs^1^ for usage rates (*Table 1*). These costs were multiplied by the number of calls averted to determine overall PPE cost savings. The cost savings of PPE used were then added to the activity costs to determine final cost savings (*Fig. 1*).

**Table 1.** Personal Protective Equipment Costs^1^ and Usage Rates

ITEM	USAGE RATE	UNIT COST	MEAN COST
Gloves			$0.08
Vinyl	0.50	$0.06	
Nitrile	0.50	$0.10	
Masks			$4.25
3-ply	0.30	$0.75	
N95	0.70	$5.75	
Gown	1.00	$5.00	$5.00
Face shield	10 uses/shield	$4.50	$0.45

1 Berkan, J 2020.

Only costs that are avoided due to a change in the care delivery process need to be considered when comparing the economic value of new interventions to standard practice.^9^ Thus, in the early days of the pandemic, the CVM monitors had sufficient capacity to add COVID-19 patients to their workload and, therefore, did not generate additional costs. However, as the pandemic progressed, we at times had to increase staffing. We have provided these costs separately as a sensitivity analysis because they can be considered semi-fixed cost associated with strategic capacity choices. Although the appropriate fitting of N95 masks is time consuming and resource intensive, this process must be completed whether CVM was used or not. Therefore, mask fitting and training costs were not included in our cost estimates.

## Results

### IN-PERSON INTERACTIONS AVERTED

The CVM program had early success that led to rapid scale up.^10^ Between March 16 and April 18, there were 5,042 in-person interactions averted for a mean of 153 calls per day between the CVM systems and health care professionals caring for these confirmed or suspected COVID-19 patients. Over the first 52 days of the pandemic, the use of the CVM system averted a mean of 8.9 in-person interactions per monitored patient day, or ∼8,000 calls.

This represents a significant reduction in health care worker exposure, time spent donning and doffing PPE, and conservation of a substantial amount of PPE, giving that these workers would have otherwise gone into the patient's room. Verbal responses to calls through the CVM were for patient questions, conversations with patients, such as reminders to not get out of bed, and reminders of diet orders in response to requests for food or drink. CVM system use fluctuated over the rest of 2020 as the COVID-19 patient load varied. By the end of 2020, there were 1,378 monitored patient days and a total of 19,086 COVID-19 patient calls using the CVM system. Importantly, there were no observed adverse events associated with the use of the CVM system.

### COST SAVINGS

**Fig. 2.**
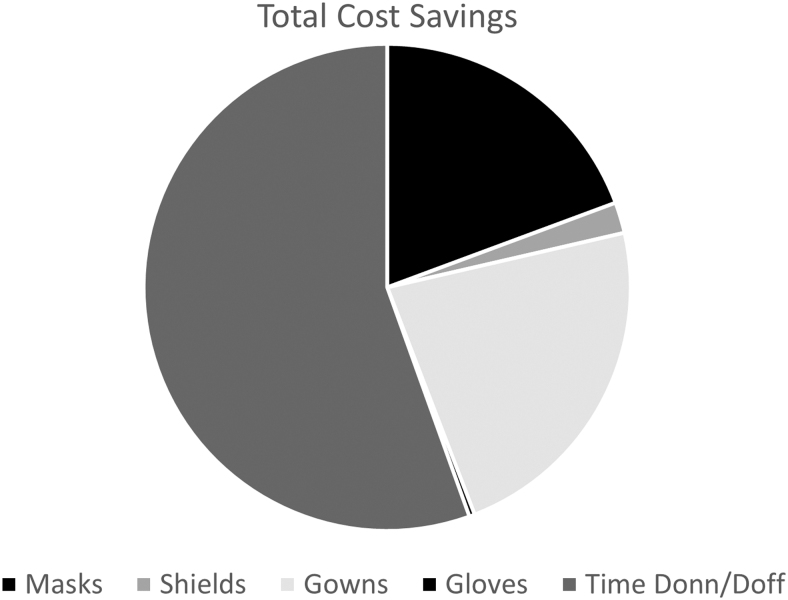
Distribution of cost savings.

The estimated cost savings for the CVM program for COVID-19 patients in 2020 were $419,319, not including potential savings from avoided COVID-19 transmissions to health care workers. A total of ∼19,000 PPE changes were avoided, with savings of $186,661 using a cost of $9.78 per PPE change based on post-COVID-19 pandemic prices for mask, gown, gloves, and face shields; $232,658 was saved in staff time from not donning (4 min) and doffing (6 min) PPE, estimated at a cost of $12.19 per call based on median nurse salary rates and the CDC-recommended PPE approach (*Fig. 2*). A total of $19,441 was spent on CVM system cleaning, maintenance, and depreciation.

Subtracting this expense from the $419,319 cost savings yields a net savings of $399,878. This net saving provides an outstanding return on investment (ROI) of 20.6 (2,060%) for the CVM program for COVID-19 patient care ($399,878/$19,441). These savings assume that we did not have to increase the number of patient monitors to meet the needs of the program. If we assume that a system needed to add 0.5 full-time equivalent for each 24-h period, the savings would be reduced. We estimate this added cost would be $15 per hour for 3,240 h, or a total of $48,600 in increased salary costs. This would reduce the expected net savings to $351,278 and change the ROI to 5.16 (or a 516% ROI).

## Discussion

In the greater context of the COVID-19 pandemic, the entire health care delivery system experienced an unanticipated shortage of skilled clinicians, essential equipment, and of PPE vital to the delivery of care. In this article, we discuss the deployment of a highly adaptive CVM system in a large academic medical center. We demonstrate that this innovative application of CVM contributed to both patient and provider safety while judiciously limiting the potential misallocation of scarce PPE resources. The reduction in patient–provider exposure represents a significant cost avoidance in the form of potential provider infection and patient cross-contamination. In addition, we demonstrated a significant cost savings originating from savings in provider time and PPE procurement, distribution, and utilization.

Particularly during the early stages of the COVID-19 pandemic, hospitals across the world made attempts to improve operational efficiency with scarce resources. A recent publication discusses the use of a vitals monitoring system that, unfortunately, did not reduce the number of patient visits nor PPE use.^11^ In this study, most of the staff were inexperienced with the CVM equipment that can lead to a lack of staff confidence.^12^

In contrast, the staff in our project had become familiar with the CVM system for high-risk behavioral patients in the year before repurposing for COVID-19 support. In addition, the previous study used vitals monitoring systems that did not have an audiovisual component. This may have led to escalating patient calls unnecessarily to in-person interactions.^12^ We feel our audiovisual capability was a crucial component to the success of our system as it enabled two-way communication to meet patient needs. Although not a part of our data collection, this may also help to reduce patients' feelings of isolation, a concern raised by the previous study.^11^

The clinical application of a CVM program for in-patient monitoring and care has potential that reaches beyond the COVID-19 pandemic. As we experience greater exposure in general to diseases such as Ebola and dengue fever, we envision several applications of an in-patient CVM system. The implementation of this system allows for the efficient use of clinician time and resources while contributing to an uninterrupted operational flow and enhancing patient–provider safety.

The substantial benefit of decreased time and exposure to infected patients by caregivers cannot be overstated. The utilization of MUSC's CVM program streamlined workflow making it more efficient and effective while reducing patient and caregiver risk. Perhaps the most obvious and immediate clinical use would be in the protection of patients who are in immunocompromised states yet require hospitalization.

Specifically, the use of the CVM program for postbone marrow and organ transplant patients could have an exponential impact both in the reduction of post-transplant infections and in cost savings in the form of clinician time and PPE use. In addition, the trajectory of this chronic yet critical patient population necessitates systems that decrease the patient's exposure to infectious pathogens while allowing for some semblance of patient autonomy and freedom.

There were limitations to this study that could be explored in future studies. We used a CDC videorecording of PPE donning and doffing to estimate time. It is possible that personnel do not follow proper procedures, in which case cost savings would decrease with a possible increase of costs due to higher infection rates. We do not have patient satisfaction/feedback data to examine the patient perspective. Patient feedback is important for any new approach to care delivery.

In addition, the use of a process improvement design means that we do not have outcomes data nor the ability to compare with a control group, which limits the strength of our findings. This was a single-center study, and our findings may not be relevant to other hospitals. We did not specifically measure quality of care. However, because nurses were still able to enter patients' rooms when the situation demanded an in-person escalation, this increases our confidence that the level of care quality was maintained.

## Conclusion

The use of CVM for in-patient populations with both confirmed and suspected COVID-19 represents an important clinical application of this emergent technology. The use of CVM in these patients and in this setting demonstrated significant cost avoidance and time savings while minimizing exposure and potential cross-infection. There is a need for further prospective research initiatives and assessment of patient perspectives as well as the potential for use of CVM in other vulnerable patient populations.
